# The effect of emotional freedom technique on premenstrual syndrome, menstrual symptoms and quality of life experienced by nursing students: a randomized controlled trial

**DOI:** 10.1007/s00737-026-01732-z

**Published:** 2026-06-19

**Authors:** Tuğba Yakıcı, Hande Yağcan

**Affiliations:** 1 Institute of Health Science, Nursing Department, Dokuz Eylül University, Izmir, Turkey; 2 Faculty of Nursing, Obstetric and Gynecologic Nursing Department, Dokuz Eylül University, Izmir, Turkey

**Keywords:** Emotional freedom technique, EFT, Menstrual symptoms, Premenstrual syndrome

## Abstract

**Purpose:**

This randomized controlled trial aimed to investigate the effect of Emotional Freedom Technique (EFT) on premenstrual syndrome, menstrual symptoms, and quality of life among nursing students.

**Methods:**

The study was conducted with 80 nursing students enrolled at XXX University, who were randomly assigned to an experimental group (*n* = 40) and a control group (*n* = 40) using a pretest–posttest design. Data were collected using the Personal Information Form, Premenstrual Syndrome Scale, Menstrual Symptom Scale, Subjective Units of Distress Scale, and the SF-12 Health Survey. The experimental group received EFT interventions, while the control group received no intervention during the study period. Pre- and post-intervention measurements were obtained for both groups.

**Results:**

Following the intervention, statistically significant differences were found between the experimental and control groups in Premenstrual Syndrome Scale, Menstrual Symptom Scale, and Subjective Units of Distress Scale and their subdimensions (*p* < 0.05). The experimental group also demonstrated significant improvements from pretest to posttest across these measures (*p* < 0.05). Regarding quality of life, the Mental Component Summary score of the SF-12 increased significantly in the experimental group (*p* < 0.05), whereas the Physical Component Summary score showed an increase that did not reach statistical significance (*p* > 0.05).

**Conclusions:**

The findings suggest that EFT may be an effective complementary intervention for reducing premenstrual and menstrual symptoms and improving mental aspects of quality of life among nursing students. EFT may be considered a potentially supportive, non-pharmacological approach in the management of menstrual-related symptoms.

**Trial registration:**

Clinical Trials.gov, NCT06557070.

## Highlights

EFT reduced premenstrual and menstrual-related distress in nursing students.

Participants reported improved emotional well-being after EFT.

Mental quality of life showed positive changes with EFT.

EFT offers a simple and non-drug supportive approach.

The findings support EFT use in women’s mental health care.

## Introduction

Premenstrual syndrome (PMS) is a condition that occurs during the luteal phase of the menstrual cycle and begins a few days before menstruation, negatively affecting women’s physical, psychological, and behavioural functioning (Gnanasambanthan and Datta 2019; The American College of Obstetricians and Gynecologists (ACOG) 2024). Its prevalence is 47.8% worldwide and 52.2% in Turkey (Erbil and Yücesoy 2022; Gudipally and Sharma 2024). Although the exact aetiology is unclear, many factors are thought to be involved, including hormonal imbalances (oestrogen, progesterone, aldosterone), thyroid dysfunction, genetic predisposition, stress, medication use, and lifestyle (Uzuner and Koçak 2019; Topatan and Kahraman 2020; Bakır and Kızılkaya Beji 2021). PMS symptoms vary from person to person, with over 200 symptoms identified. These symptoms have been shown to reduce quality of life, weaken social relationships, and negatively affect academic and professional performance (Çevik and Alan 2021; Branecka Wozniak et al. 2022; The American College of Obstetricians and Gynecologists (ACOG) 2024).

**Fig. 1:**
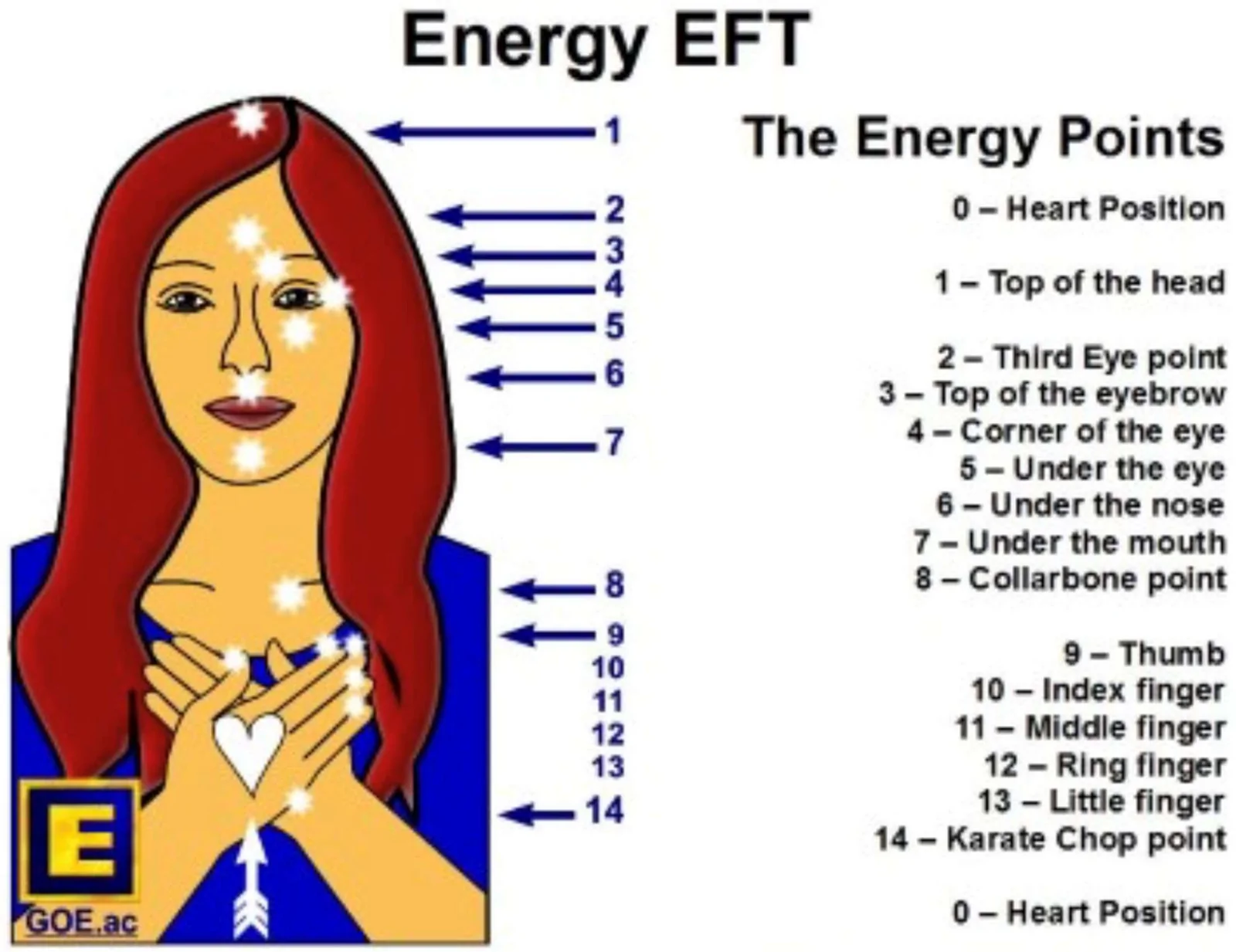
The energy points of EFT

It has been reported that women use many pharmacological and non-pharmacological methods to cope with PMS. Herbal teas, vitamin supplements, massage, heat application, yoga, meditation, prayer, acupressure and similar approaches are frequently preferred (Eshetu et al. 2022; Şimşek et al. 2022). One such method, the Emotional Freedom Technique (EFT), is based on tapping specific points on the energy meridians surrounding the body with the fingertips Fig. 1 (Hartmann 2016; Rancour 2017; Vural and Aslan 2018; Altuntaş and Düzgüner 2020).

**Fig. 2:**
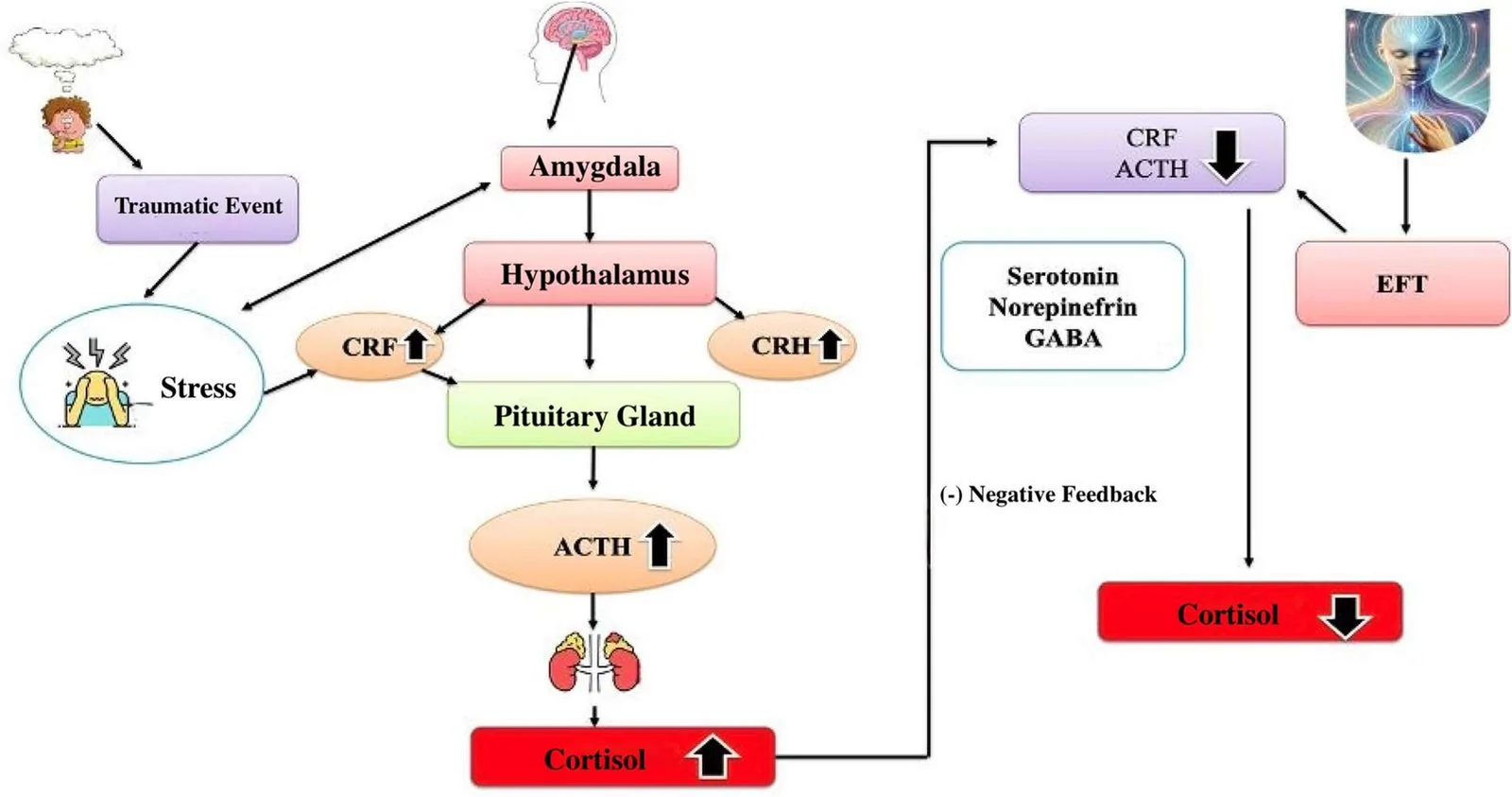
The Relationship Between EFT and the Stress Feedback Mechanism

In the limbic system, the amygdala, located at the centre of the emotional brain, determines whether an incoming emotion is a threat, connects with the hypothalamus, and activates the sympathetic nervous system (Greenberg 2004; Zhu et al. 2018). When the brain encounters any stressor, it releases corticotropin-releasing hormone (CRF) from the hypothalamus. The release of this hormone stimulates the pituitary gland to release Adrenocorticotropic Hormone (ACTH). The stimulated adrenal glands increase cortisol release (Thau et al. 2024). When cortisol levels accumulate excessively, negative feedback via CRH, serotonin, norepinephrine, gamma-aminobutyric acid (GABA), or endogenous opioids decreases CRF and ACTH levels, and once cortisol levels are regulated, stress levels are also regulated Fig. 2 (Guyton 2013; Feinstein 2019).

However, in scenarios where stress is not controlled, physical and psychological adverse outcomes may occur (Sebastian and Nelms 2017; Bach et al. 2019; Stapleton et al. 2020).

EFT has been reported to be effective in managing psychological problems such as stress, anxiety, and depression. Due to its reliability and lack of side effects have become a preferred method for various ages and groups (Church et al. 2018; Vural and Aslan 2018). One study found that EFT can reduce fear of childbirth, while another found that it can reduce nurses’ stress and burnout levels in a single session during the pandemic (Irmak Vural et al. 2019; Dincer and Inangil 2021).

The adverse effects of PMS on quality of life have been frequently emphasised in the literature, particularly in young women, with findings such as decreased academic achievement, loss of productivity, and reduced self-confidence(Toptaş Acar). An acupressure-based study reported a reduction in PMS symptoms and an increase in quality of life (Şimşek Küçükkelepçe and Timur Tashan 2021). Yoga and similar relaxation techniques have also been shown to positively affect PMS symptoms (Şimsek Küçükkelepçe et al. 2021). Similarly, although studies on EFT are limited, existing studies suggest that it may positively alleviate PMS symptoms (Bakır et al. 2022; Yazar et al. 2025; Özşahin et al. 2025).

EFT is considered a low-cost method that individuals can apply, making it a potentially noteworthy alternative for managing PMS symptoms (Rancour 2017; Bakır et al. 2022). However, according to research, more randomised controlled trials and meta-analyses are needed to achieve stronger evidence regarding the effectiveness of EFT (Clond 2016). In line with this, our study aims to evaluate the effect of EFT on premenstrual and menstrual symptoms among nursing students experiencing PMS and to determine whether it improves their quality of life.

### Research hypotheses

- H1: EFT application reduces premenstrual syndrome in nursing students experiencing PMS compared to nursing students who did not receive the application.
- H2: EFT application reduces the severity of menstrual symptoms in nursing students experiencing PMS compared to nursing students who did not receive the application.
- H3: EFT application improves the quality of life of nursing students experiencing PMS compared to nursing students who did not receive the application.
- H4: EFT application reduces Subjective Units of Distress Scale (SUDS) scores in nursing students experiencing PMS compared to nursing students not receiving the application.

## Materials and methods

### Study design

The study is a randomised controlled, experimental and longitudinal study. It was conducted according to the Updated Guidelines for Reporting Randomised Parallel-Group Trials (Consolidated Standards of Reporting Trials-CONSORT) report (Schulz et al. 2010). The study is registered on ClinicalTrials.gov (Id No: NCT06557070).

### Study participants and setting

**Fig. 3:**
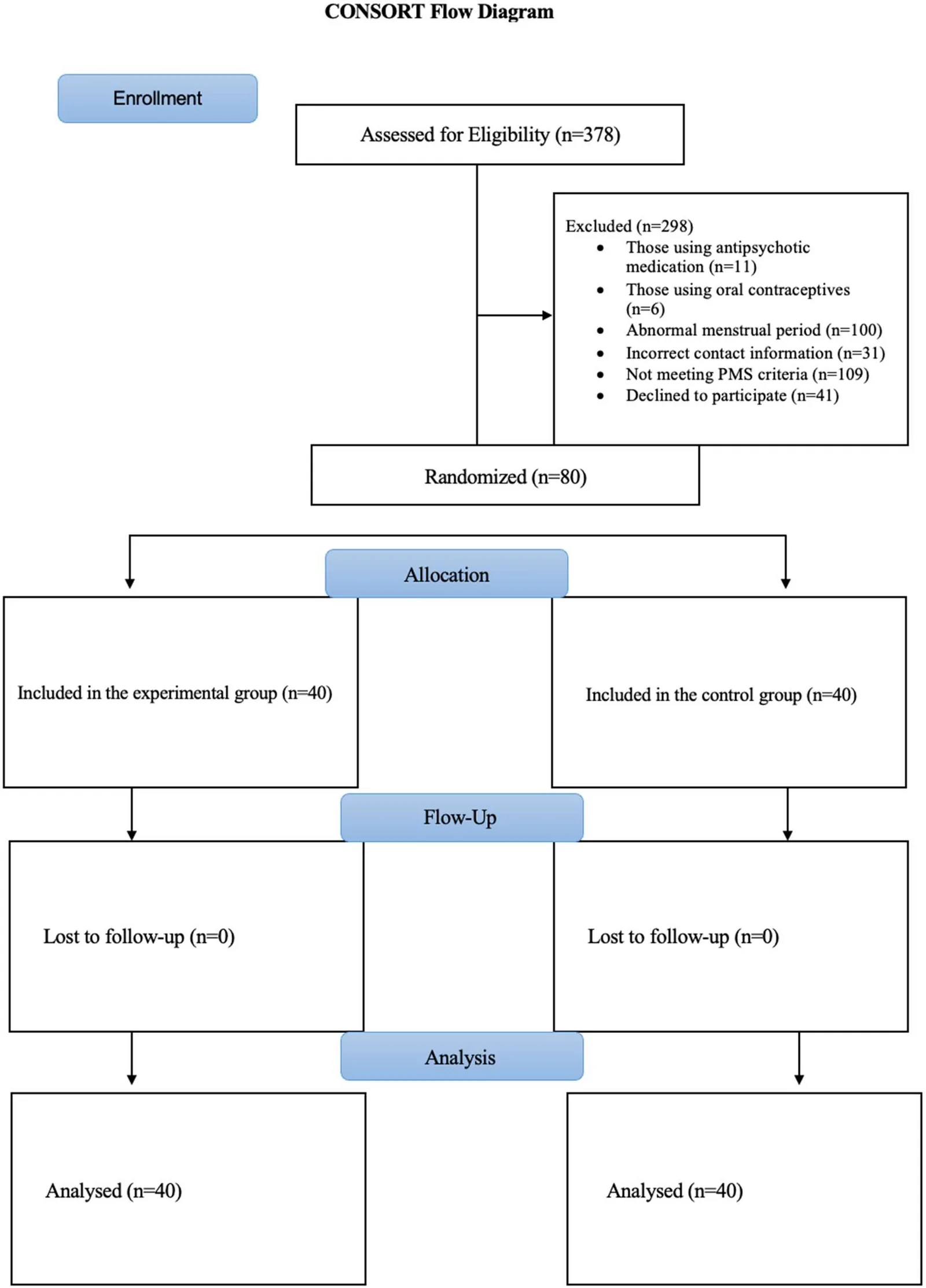
CONSORT diagram

The study was conducted with students at a university’s nursing faculty in the Aegean Region of Turkey. The study consisted of female students who met the selection criteria and volunteered. The G.Power 3.1 programme determined the number of experimental and control groups. The calculation was based on a previous study, resulting in 35 experimental group participants and 35 control group participants, for a total of 70 students, with 80% power and 95% confidence interval^42^. To prevent potential data loss, 80 female students who met the inclusion criteria were grouped into 40 experimental and 40 control groups Fig. 3 CONSORT diagram. In experimental studies, keeping losses below 10% or 15% is considered essential for reducing bias, and it is emphasised that increasing the sample size is helpful in controlling bias associated with losses (Montori and Guyatt 2001; Faltin and Ruggeri 2012; Akın and Koçoğlu 2017).

**Fig. 4:**
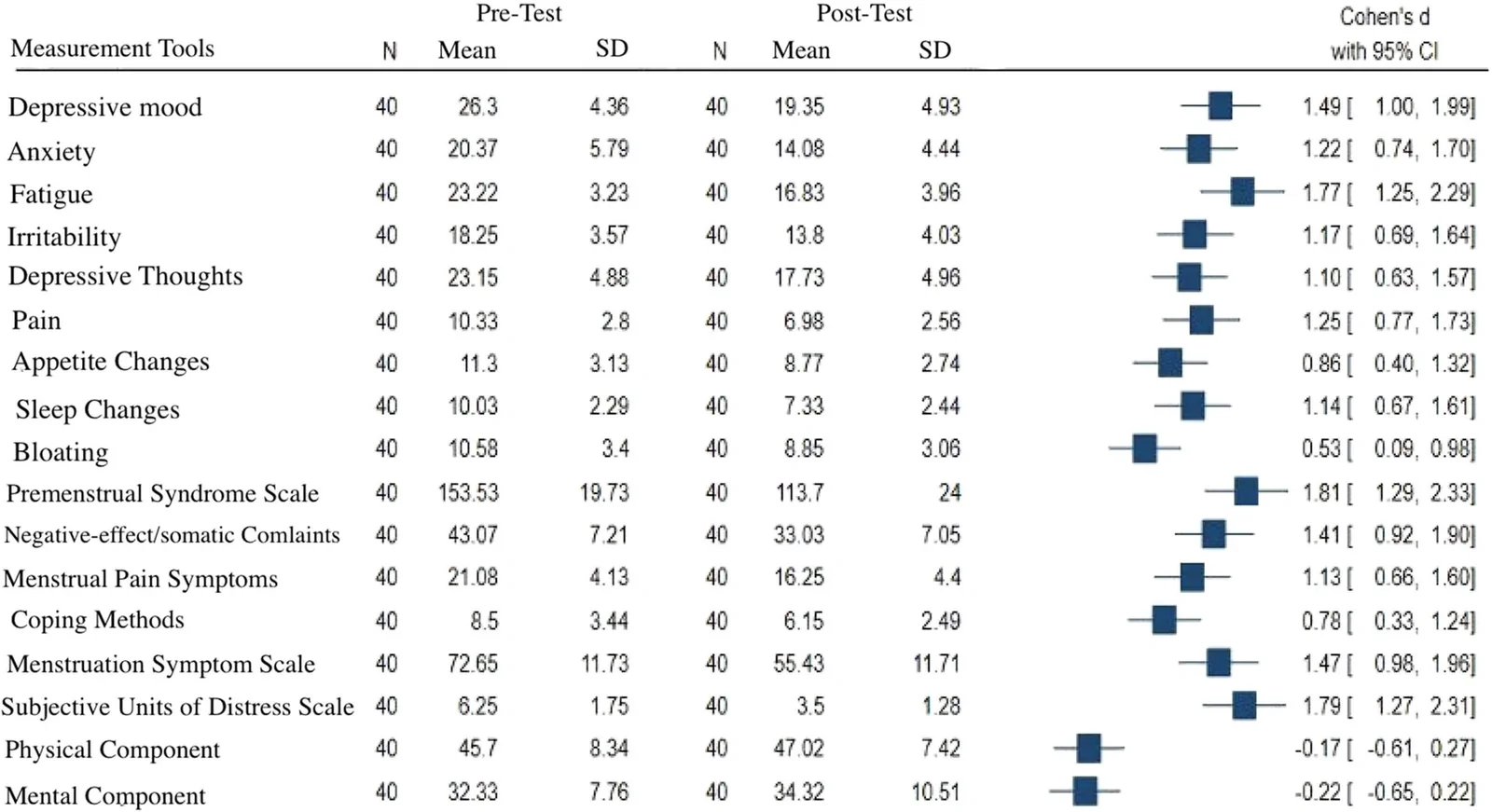
Effect Size (Experimental Group Pre-test-Post-test)

The effect size for the differences between the pre-test and post-test in the experimental group is presented in Fig. 4.

The study included female students who scored 132 or higher on the PMSS, were aged 18 or older, had a menstrual cycle length within normal limits (every 21–35 days) over the past 3 months, were volunteers, and had a smartphone and internet access. Students receiving psychiatric treatment, those who had started antidepressant medication in the last three months, and those using oral contraceptives were excluded from the study.

### Randomization and blinding

A simple randomisation method was used to assign participants to the experimental and control groups. The researchers collected the pre-test measurements of the participants to be included in the study, and an independent statistician assigned IDs and sequence numbers to the relevant individuals. Group allocation was performed using Randomizer.org through a lottery method by an independent statistician in a blind manner. Allocation concealment was ensured, as the researchers responsible for data collection were not involved in generating the randomisation sequence. The students included in the study did not know which group they were in. Furthermore, an independent statistician conducted the survey analysis (Schulz and Grimes 2002; Schulz et al. 2010).

Due to the behavioural nature of the intervention, blinding of participants was not feasible. However, several measures were implemented to reduce potential bias. The statistician who performed the data analysis was blinded to group allocation. Data were coded prior to analysis to prevent identification of experimental or control groups. As all outcome measures consisted of validated self-administered scales, no external outcome assessor was involved. To minimise contamination between groups, several precautions were taken. Although both groups were recruited from the same faculty, intervention sessions were conducted individually (one-to-one) and scheduled at different times based on participants’ availability. A separate WhatsApp group was created exclusively for the experimental group to share EFT-related materials, session reminders, and affirmations. The control group did not have access to these materials during the study period. Participants were explicitly instructed not to share intervention content with other students until the study was completed (Torgerson^2001^; Schulz et al. 2010).

### Control condition

The control group received a standardised face-to-face educational session about premenstrual syndrome and menstrual health but did not receive the EFT intervention during the eight-week study period. This approach was used to control for potential informational effects while isolating the specific effect of EFT. After completion of post-test assessments, EFT training was offered to the control group to ensure ethical balance (Togerson 2001; Schulz et al. 2010).

### Data collection

#### Personal information form

The researcher created this form using relevant literature to determine the participants’ sociodemographic characteristics, lifestyle, and menstrual history and ensure a homogeneous randomisation distribution. It consists of 21 questions (Vural and Aslan 2018; Uzuner and Koçak 2019; Topatan and Kahraman 2020; Bakır and Kızılkaya Beji 2021; Bakır et al. 2022).

#### Premenstrual syndrome scale (PMSS)

This scale, developed by Gençdoğan (2006), is a 44-item, five-point Likert-type measurement tool designed to identify and assess the severity of premenstrual symptoms. The “Never” option is scored 1 point, “Very Rarely” 2 points, “Sometimes” 3 points, “Often” 4 points, and “Always” 5 points. The scale measures depressive mood (1,2,3,4,5,6,7), anxiety (8,9,10,11,13,15,16), fatigue (12,14,18,25,37), irritability (19,20,21,22,23), depressive thoughts (24, 26, 27, 28, 29, 30, 44), pain (31, 32, 33), appetite changes (34, 35, 36), sleep changes (38, 39, 40), and bloating (41, 42, 43). The scores obtained from each sub-dimension can be evaluated separately, and the “PMSS Total Score” is obtained from the sum of all sub-dimensions. The total score range for the scale is 44–220. High scores indicate more intense premenstrual syndrome symptoms. The Cronbach’s Alpha value for the scale was 0.75. Additionally, the Cronbach’s Alpha values of the subscales range from 0.75 to 0.91 (Gençdoğan 2006).

In this study, Cronbach’s alpha for this scale was 0.908 in the pre-test and 0.969 in the post-test. The Cronbach’s Alpha values for the subscales ranged from 0.742 to 0.880 for the pre-test and from 0.795 to 0.930 for the post-test.

#### SF-12 health survey

Ware and colleagues (1995) developed the SF-12, which contains the same subscales as the SF-36, to determine quality of life. Similar to the SF-36, it consists of 8 subscales and 12 items: physical functioning (2,3), physical role (4,5), bodily pain (8), general health (1), energy (10), social functioning (12), emotional role (6,7), and mental health (9,11). Items related to physical and emotional roles are answered with yes or no, while other items have Likert-type options ranging from 3 to 6. The Physical Component score is obtained from the sub-dimensions of general health, physical functioning, physical role, and body pain. In contrast, the Mental Component score is obtained from the social functioning, emotional role, and mental. Health and energy sub-dimensions. Both component scores range from 0 to 100, with higher scores representing better quality of life (2024). Soylu and Kütük (2022) conducted a Turkish validity and reliability study in healthy individuals and found a Cronbach’s Alpha value of 0.72 (Soylu and Kutuk 2021).

In this study, Cronbach’s alpha for this scale was 0.785 in the pre-test and 0.832 in the post-test.

#### Menstrual symptom scale (MSS)

This scale, developed in 1975 by Chesney and Tasto, assesses menstrual pain and symptoms. It was re-evaluated and updated in 2009 by Negriff and colleagues. The scale was adapted into Turkish by Güvenç and colleagues in 2014. Participants are asked to rate the symptoms they experience related to menstruation on a scale from 1 (never) to 5 (always). The scale items are numbered according to the factors they represent for ease of use. Items 1–13 belong to the “Negative effects/somatic complaints” subscale, items 14–19 belong to the “Menstrual pain symptoms” subscale, and items 20–22 belong to the “Coping methods” subscale. It is a 5-point Likert-type scale with 22 items. The subscale score is calculated as the average of the items’ total scores within the subscale. An increase in the subscale mean score indicates an increase in the severity of menstrual symptoms related to that subscale. The Cronbach’s Alpha value is 0.86 (Guvenc et al. 2014).

In this study, Cronbach’s alpha for this scale was 0.863 in the pre-test and 0.944 in the post-test. The Cronbach’s Alpha values for the sub-dimensions ranged from 0.684 to 0.814 in the pre-test and from 0.766 to 0.912 in the post-test.

#### Subjective units of distress scale (SUDS)

Developed by Wolpe in 1973, the subjective assessment involves the individual evaluating the discomfort they feel in response to the stimulus causing their anxiety, scoring “0” for no discomfort and “10” for unbearable pain on a scale of 0 to 10. A high score indicates that the individual feels a high level of anxiety or stress (Wolpe 1973).

#### Menstrual cycle and EFT session participation tracking form

The researcher developed this form to track students’ menstrual cycle patterns and monitor their attendance at EFT sessions.

### Data collection

**Fig. 5:**
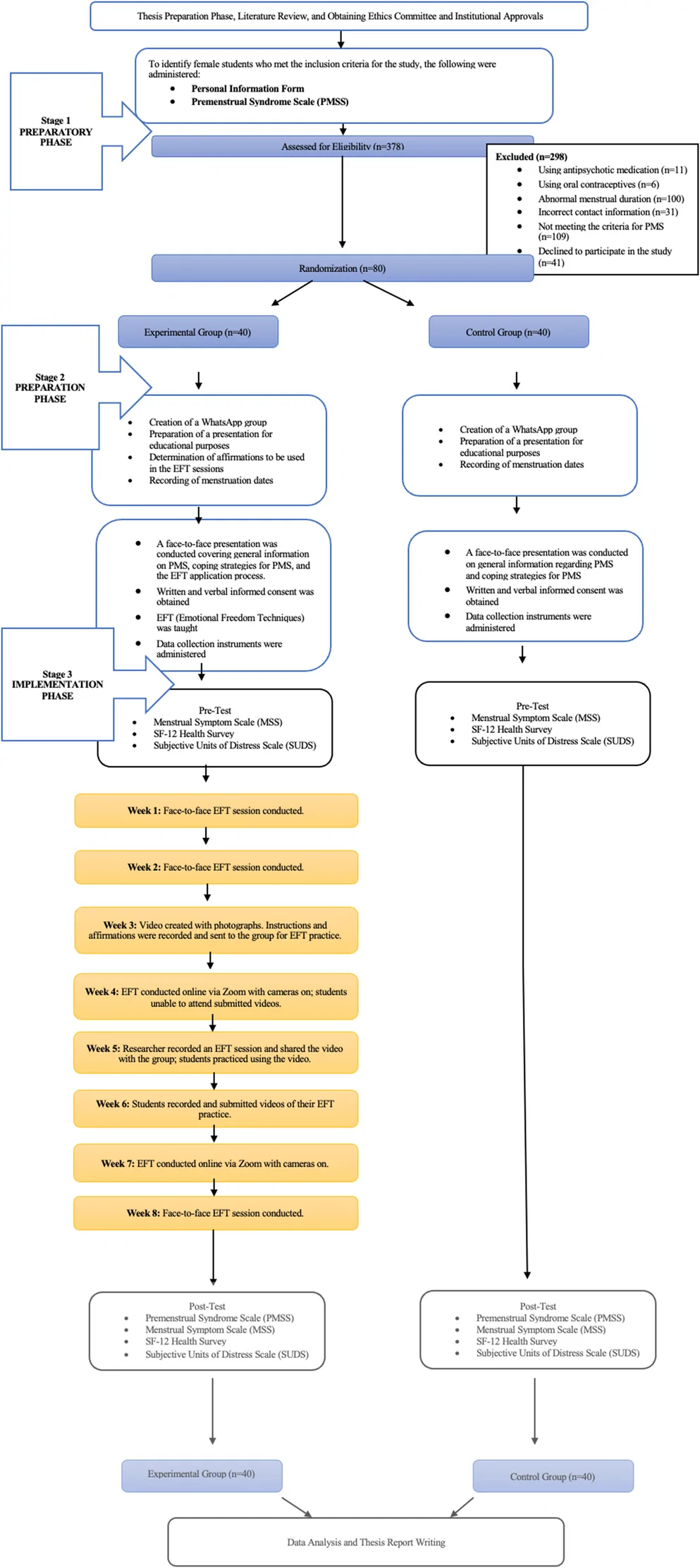
Research Flow Chart

The research was conducted in three phases: pre-preparation, preparation, and implementation. The implementation flowchart is shown in Fig. 5.

During the implementation phase, students in the control group gathered at the XXX University Faculty of Nursing’s pregnancy education classroom on the selected days and times, and a presentation on PMS was given. Information was provided that the study would last for two menstrual cycles. Students were asked not to use analgesics during the study period and to report if they did. At the end of the implementation phase, the EFT application was explained to the students, and they were taught how to perform it, in accordance with the principle of equality.

The experimental group gathered at the XXX Faculty of Nursing antenatal education class on the day and time chosen by the students. The students attended whichever session was scheduled for them that week. First, a presentation containing information about PMS and EFT was given to the students. Information about the research was provided. It was stated that the application would last for two cycles, that the applications should be performed once a week, and that the researcher would follow up. The decision to carry out the application in this manner was made based on the training received by the researcher and studies conducted in the literature. The students’ participation in the training sessions was recorded weekly in a table. The EFT session began with the researcher demonstrating the application points on themselves. Subsequently, EFT was applied to the students individually. They were asked to perform EFT applications on themselves under the researcher’s supervision, and any application errors were corrected. Finally, the students’ EFT applications to one another were observed, and the day’s session concluded. Luteal phase days were calculated based on previously recorded menstruation days, and application weeks were initiated. Students whose weeks aligned were included in the EFT application meetings together. In subsequent weeks, each student continued to attend the application meetings on a day suitable for them. The EFT application meetings were held every week on days and at times convenient for the students, determined by survey results from WhatsApp groups. The sessions were conducted one-on-one. At the beginning of each week, affirmations were sent to students to motivate and boost their self-esteem. The SUE scale was applied before and after the EFT sessions.

Additionally, all students participating in the research were given a small gift.

### EFT application steps

- ***Heart Healing Position:*** This is the position known as the starting point of the EFT round, where both hands are placed on top of each other directly over the heart. Students were asked to assume the heart healing position and take three deep breaths with their eyes closed.
- ***Identifying the Problem:*** The focus was on the students' experiences with premenstrual syndrome symptoms.
- ***Creating a Reminder Phrase (Emotional Statement):*** This is a short sentence that helps express the emotion experienced so that the problem can be remembered later. To ensure focus, students were asked to repeat it aloud. The clearer, more direct, and more realistic the setup sentence is, the greater the change achieved(Hartmann 2016). An example of a reminder sentence could be: "Even though my body tells me it's at its peak with PMS pain, headaches, or cramps, I respectfully accept my body."
- ***Assessment of the Problem:*** Students were asked to think about their premenstrual syndrome symptoms and rate their feelings on the SUE scale.
−10 represents the greatest possible pain, disappointment, fear, stress, sadness, or discomfort.
- +10 represents the highest level of joy, happiness, or feeling wonderful.
- ***Performing the Tapping Sequence:***
- The taps were generally performed using the middle and index fingers.
- Each point was tapped at least seven times.
- The taps are performed firmly enough to create awareness and focus, but not too hard.
- Everyone's tapping does not have to be the same; what matters is the location being tapped.
- Affirmations were recited aloud while the tapping was being performed.
- The tapping procedure was performed sequentially on 14 acupressure points (crown of the head, third eye point, eyebrow start, eye corner, under the eye, under the nose, under the lip, under the collarbone, thumb, index finger, middle finger, ring finger, little finger, karate point).
- ***Creation of Suggestions:*** These are suggestions created to make students feel strong and positive. An example is: "Even if I don't want to talk to anyone, even if I want to stay in bed, I know and accept that this is a normal part of my period."

### Statistical analysis

The data obtained in the study were analysed using SPSS (Statistical Package for the Social Sciences) for Windows 25.0. Descriptive statistical methods (number, percentage, minimum, maximum, mean, standard deviation) were used to analyse the data.

The normality of the data distribution was tested using the Shapiro-Wilk test. The distribution’s normality can be examined using a Q-Q plot. Furthermore, the normality of the data distribution depends on the skewness and kurtosis values being within ± 2 (George and Mallery 2010). The normality of the data distribution was assessed using skewness and kurtosis.

In data showing a normal distribution, the t-test (independent sample t-test) was used for the comparison of two independent groups, and the dependent group t-test was used for two.

related measurements. The Levene statistic was used to determine the homogeneity of variance. The relevant test was used to determine whether the variances were homogeneous.

Regression analysis was performed to explain changes in the scores on the scales used. A significance level of *p* < 0.05 was used to determine whether the obtained values were significant. In addition, the chi-square test was used to examine whether the groups showed similar distributions.

## Results

The distribution and comparison of students’ sociodemographic and menstruation characteristics according to groups are presented in Table 1. No statistically significant differences were found between the experimental and control groups regarding socio-demographic and menstrual characteristics at baseline (*p* > 0.05), indicating that the groups were homogeneous before the intervention.

**Table 1:** Distribution and comparison of students’ socio-demographic and menstruation characteristics according to groups (*n* = 80)

		Group				Test Statistic Value	p
		Experiment		Control			
		n	%	n	%		
Age	20 years old and under	25	62.5	21	52.5	0.818^P^	0.366
	21 years and older	15	37.5	19	47.5		
BMI	Underweight	3	7.5	4	10.0	3.411^FE^	0.301
	Normal weight	27	67.5	32	80.0		
	Overweight	9	22.5	4	10.0		
	Obese	1	2.5	0	0		
Attendance at antenatal classes	Not taking antenatal classes	22	55.0	21	52.5	0.050^P^	0.823
	Womenwhotook childbirth classes	18	45.0	19	47.5		
Marital status	Single	40	100.0	40	100.0	----	----
	Married	0	0.0	0	0.0		
Place of residence	Country	28	70.0	23	57.5	1,667^FE^	0.447
	Living with family	9	22.5	11	27.5		
	In a separate home	3	7.5	6	15.0		
Income status	Income is less than expenditure	18	45.0	18	45.0	0.101^P^	0.951
	Revenueequals expenditure	15	37.5	14	35.0		
	Incomeexceeds expenditure	7	17.5	8	20.0		
Health status	Poor	2	5.0	1	2.5	1.202^FE^	0.599
	Medium	27	67.5	24	60.0		
	Good	11	27.5	15	37.5		
Age at First Menstruation	11 years and under	3	7.5	3	7.5	6.063^FE^	0.184
	12 years	18	45.0	11	27.5		
	13 years old	9	22.5	15	37.5		
	14 years old	10	25.0	8	20.0		
	15 years and older	0	0.0	3	7.5		
Menstrual cycle	Regular (21–35)	40	100.0	40	100.0	-----	----
	Normal	0	0.0	0	0.0		
Menstrual Period	2–6 days	35	87.5	33	82.5	0.392^P^	0.531
	≥ 7	5	12.5	7	17.5		
How is your menstrual period going?	Painful	29	72.5	29	72.5	0.000^P^	1.000
	Painless	11	27.5	11	27.5		
		Group				Test Statistic Value	p
		Experiment		Control			
		n	%	n	%		
Have you consulted a healthcare facility regarding PMS?	Yes	9	22.5	11	27.5	0.267^P^	0.606
	No	31	77.5	29	72.5		
What method do you use to manage PMS?	I don’t use anything	8	20.0	7	17.5	3.228^P^	0.358
	Lifestyle (walking, exercise, diet)	3	7.5	8	20.0		
	Complementary therapies (vitamins, minerals, herbal teas, etc.)	11	27.5	7	17.5		
	Medical treatment (pain reliever)	18	45.0	18	45.0		
Your Diet	High in fat, sugar, and salt	19	47.5	19	47.5	6.122^FE^	0.097
	Vegetables, fruit	11	27.5	13	32.5		
	Meat, poultry, fish	7	17.5	1	2.5		
	Tableware	3	7.5	7	17.5		
Your exercise status	Never	8	20.0	6	15.0	0.518^FE^	0.868
	Occasionally	29	72.5	30	75.0		
	Usually (at least 3 days a week for 30 min)	3	7.5	4	10.0		
Do you have any sleep problems?	Yes	13	32.5	12	30.0	0.058^P^	0.809
	No	27	67.5	28	70.0		
Do you use addictive substances (alcohol/tobacco, etc.)?	Yes	7	17.5	8	20.0	0.082^P^	0.775
	No	33	82.5	32	80.0		
Do you consume more than two cups of coffee a day?	Yes	16	40.0	17	42.5	0.052^P^	0.820
	No	24	60.0	23	57.5		
History of PMS in mother or sister	Yes	12	30.0	13	32.5	0.058^P^	0.809
	No	28	70.0	27	67.5		
If so, who has it?	Sister	3	25.0	6	46.2	1.619^FE^	0.464
	Mother	5	41.7	5	38.5		
	Both	4	33.3	2	15.4		
Are you using birth control pills?	Yes	0	0.0	0	0.0	----	----
	No	40	100.0	40	100.0		
Have you started taking psychiatric medication in the last three months?	Yes	0	0	0	0.0	----	----
	No	40	100.0	40	100.0		

*FE* Fisher’s Exact Test, *P* Pearson chi-square test

The comparison of the mean scale total and subscale scores between groups and within groups based on the pre-test and post-test measurements of the students in the experimental and control groups is presented in Table 2. Baseline pre-test scores did not differ significantly between the experimental and control groups across all scales, indicating comparable group characteristics before the intervention (*p* > 0.05). Following the intervention, the experimental group demonstrated significantly lower total PMSS scores and all PMSS subscale scores, including depressive mood, anxiety, fatigue, irritability, depressive thoughts, pain, appetite changes, sleep changes, and bloating, compared with the control group (*p* < 0.05). Within-group analyses also showed significant reductions in all PMSS subscales and total scores in the experimental group, whereas the control group showed limited improvement only in depressive mood, irritability, and pain subscales.

Similarly, post-test MSS total and subscale scores (negative effects/somatic complaints, menstrual pain symptoms, and coping methods) were significantly lower in the experimental group than in the control group (*p* < 0.05). Significant pre–post improvements were observed across all MSS domains in the experimental group, while no significant changes were detected in the control group.

For the SUDS, no baseline difference was found between groups; however, post-test scores significantly favored the experimental group (*p* < 0.05). The experimental group showed a significant reduction in distress levels, whereas no significant change occurred in the control group. Regarding quality of life outcomes, SF-12 physical component scores did not significantly differ either between or within groups (*p* > 0.05). In contrast, the SF-12 mental component score differed significantly at post-test, with lower scores observed in the control group (*p* < 0.05). Within-group analysis revealed a significant decline in mental component scores in the control group, while the experimental group maintained stable mental health scores.

**Table 2:** Comparison of the mean total scale and subscale scores between groups and within groups based on the pre-test and post-test measurements of the students in the experimental and control groups

		Group		t-test^2^	*p*-value
		Experimental1	Control		
		Mean ± SD	Mean ± SD		
Premenstrual Syndrome Scale	Pre-test	153.53 ± 19.73	160.5 ± 21.41	−1.515	0.134
	Final test	113.7 ± 24	149.43 ± 24.71	−6.559	0.000*
t-test^1^		11.071	2.636		
p-value		0.000*	0.012		
Depressive Mood	Pre-test	26.3 ± 4.36	27.78 ± 4.99	−1.408	0.163
	Final test	19.35 ± 4.93	25.45 ± 4.28	−5.908	0.000*
t-test^1^		8.879	2.780		
p-value		0.000*	0.008*		
Anxiety	Pre-test	20.38 ± 5.79	22.2 ± 5.43	−1.455	0.150
	Final test	14.08 ± 4.44	19.95 ± 5.62	−5.185	0.000*
t-test^1^		6.166	1.963		
p-value		0.000*	0.057		
Fatigue	Pre-test	23.23 ± 3.23	23.53 ± 4.06	−0.366	0.715
	Final test	16.83 ± 3.96	22.05 ± 3.82	−6.009	0.000*
t-test^1^		10.573	1.977		
p-value		0.000*	0.055		
Irritability	Pre-test	18.25 ± 3.57	19.23 ± 3.82	−1.179	0.242
	Final test	13.8 ± 4.03	17.28 ± 4.13	−3.812	0.000*
t-test^1^		7.635	2.602		
p-value		0.000*	0.013*		
Depressive Thoughts	Pre-test	23.15 ± 4.88	24.15 ± 5.88	−0.828	0.410
	Final test	17.73 ± 4.96	23.63 ± 5.24	−5.173	0.000*
t-test^1^		6.645	0.500		
p-value		0.000*	0.620		
Pain	Pre-test	10.33 ± 2.8	11.1 ± 3.14	−1.163	0.248
	Final test	6.98 ± 2.56	9.65 ± 2.65	−4.598	0.000*
t-test^1^		9.330	3.172		
p-value		0.000*	0.003*		
		Group		t-test^2^	*p*-value
		Experimental1	Control		
		Mean ± SD	Mean ± SD		
Appetite Changes	Pre-test	11.3 ± 3.13	11.83 ± 3.09	−0.755	0.452
	Final test	8.78 ± 2.74	11.05 ± 2.71	−3.735	0.000*
t-test^1^		4.408	1.714		
p-value		0.000*	0.094		
Sleep Changes	Pre-test	10.03 ± 2.29	10.48 ± 3.22	−0.720	0.474
	Final test	7.33 ± 2.44	9.6 ± 2.61	−4.025	0.000*
t-test^1^		7.087	1.706		
p-value		0.000*	0.096		
Bloating	Pre-test	10.58 ± 3.4	10.8 ± 3.07	−0.310	0.757
	Final test	8.85 ± 3.06	10.78 ± 2.62	−3.024	0.003*
t-test^1^		3.809	0.063		
p-value		0.000*	0.950		
Menstrual Symptom Scale	Pre-test	72.65 ± 11.73	76.03 ± 12.16	−1.263	0.210
	Final test	55.43 ± 11.71	79.13 ± 12.2	−8.864	0.000*
t-test^1^		8,790	−2.139		
p-value		0.000*	0.039		
Negative effects/somatic complaints	Pre-test	43.08 ± 7.21	44.88 ± 7.54	−1.092	0.278
	Final test	33.03 ± 7.05	46.5 ± 7.66	−8.186	0.000*
t-test^1^		8.561	−1.643		
p-value		0.000*	0.108		
Menstrual pain symptoms	Pre-test	21.08 ± 4.13	22.65 ± 4.22	−1.686	0.096
	Final test	16.25 ± 4.4	23.55 ± 3.59	−8.131	0.000*
t-test^1^		6.576	−1.796		
p-value		0.000*	0.080		
Coping methods	Pre-test	8.5 ± 3.44	8.5 ± 2.92	0.000	1.000
	Final test	6.15 ± 2.49	9.08 ± 2.85	−4.891	0.000*
t-test^1^		5.026	−1.492		
p-value		0.000*	0.144		
Subjective Units of Distress Scale		Pre-test	6.25 ± 1.75	6.05 ± 1.71	0.517
		Final test	3.5 ± 1.28	5.68 ± 1.33	−7.455
t-test^1^			11.233	1.549	
p-value			0.000*	0.129	
Physical component		Pre-test	45.7 ± 8.34	44.65 ± 7.67	0.589
		Final test	47.02 ± 7.42	44.9 ± 7.32	1.286
t-test^1^			−0.791	−0.220	
p-value			0.434	0.827	
Mental component		Pre-test	32.33 ± 7.76	35.47 ± 9.26	−1.641
		Final test	34.32 ± 10.51	29.51 ± 8.65	2.236
t-test^1^			−1.199	3.232	
p value			0.238	0.002*	

t test1 T test in dependent groups, t test2 T test in independent groups, *p<0.05

The findings of the dummy variable linear regression analysis conducted to determine the effect of EFT on the total scale scores obtained are presented in Table 3. Dummy variable linear regression analyses demonstrated that the EFT intervention had a significant effect on premenstrual symptoms, menstrual symptoms, subjective distress, and mental quality of life outcomes. The regression model for total PMSS scores was statistically significant (F = 27.016, *p* < 0.001), with EFT explaining 24.8% of the variance in score changes (Adj. R²=0.248). The experimental group showed a greater reduction in PMSS scores compared with the control group (β=−28.750, *p* < 0.001).

Similarly, the regression model for MSS total scores was significant (F = 69.530, *p* < 0.001), explaining 46.5% of the variance (Adj. R²=0.465). Menstrual symptom scores decreased more substantially in the experimental group than in the control group (β=−20.325, *p* < 0.001).

For SUDS scores, the model was also statistically significant (F = 47.596, *p* < 0.001), with EFT accounting for 37.2% of the variance in score changes (Adj. R²=0.372). Subjective distress levels declined significantly more in the experimental group (β=−2.375, *p* < 0.001). In contrast, the regression model for the SF-12 physical component was not statistically significant (F = 0.279, *p* > 0.05), indicating no significant effect of EFT on physical quality of life. However, the model for the SF-12 mental component was significant (F = 10.256, *p* < 0.001), explaining 10.5% of the variance (Adj. R²=0.105). Mental quality of life outcomes were significantly more favorable in the experimental group (β = 7.949, *p* = 0.002).

**Table 3:** Dummy variable linear regression analysis conducted to determine the effect of eft on total scale scores

Unstandardised			Standardised	t	p value	F value	p value	Adj. R2	DW
Beta		Std. Error	Beta						
Constant	-11.075	3.911		- 2.832	0.006	27.016	0.000	0.248	2.231
Group=Experimental	-28.750	5.531	-0.507	- 5.198	0.000				
Dependent variable: Premenstrual Syndrome Scale final test - Premenstrual Syndrome Scale pre-test									
Constant	3.100	1.724		1.799	0.076	69.530	0.000	0.465	1.620
Group=Experimental	-20.325	2.437	-0.687	- 8.338	0.000				
Dependent variable: Menstrual Symptom Scale final test - Menstrual Symptoms									
Constant	-0.375	.243		-1.541	0.127	47.596	0.000	0.372	2.149
Group=Experimental	-2.375	.344	-0.616	-6.899	0.000				
Dependent variable: Subjective Units of Distress Scale final test - Subjective Units of Distress Scale pre-test									
Constant	0.251	1.425		0.176	.861	0.279	>0.05	-0.009	1.784
Group=Experimental	1.065	2.015	0.060	0.528	.599				
Dependent variable: Physical component final test - Physical component preliminary test									
Fixed	-5.954	1.755		- 3.392	.001	10.256	0.000	0.105	2.137
Group=Experimental	7.949	2.482	0.341	3.202	.002				
Dependent variable: Mental component post-test - Mental component pre-test									

## Discussion

This study examined the effect of EFT application on the quality of life of nursing students experiencing PMS and their premenstrual syndrome and menstrual symptom experiences. In our study, there was no statistically significant difference in total and subscale scores of the PMSS between the experimental and control groups before the EFT application (Table 2; *p* > 0.05). In this case, the experimental and control groups showed similar characteristics.

EFT application statistically significantly reduced the students’ total PMSS scores (Table 2; *p* < 0.05). It was also found to reduce the mean scores of the PMSS subdimensions (Table 2; *p* < 0.05). Studies on the effects of EFT on PMS are limited. In randomised controlled trials, EFT application reduced the total PMSS score, similar to our study (Bakır et al. 2022; Özşahin et al. 2025). According to a regression analysis in an RCT, a 30.7% reduction in PMSS scores was observed, whereas in our study, this rate was 46.5% (Bakır et al. 2022). The reason why the EFT we applied in our study reduced the students’ total PMSS score more than other studies is thought to be due to the large sample size in our study, the fact that the applications were carried out under the control of the researchers, the completion of two menstrual cycles and two luteal phases, the manner in which the applications were carried out (group, individual, self- administered, peer-administered), offering different application options (online Zoom video conferencing, video recording, audio recording, face-to-face meetings).

Our study findings suggest that EFT may also reduce premenstrual syndrome symptoms. Some studies have suggested that the touch applied in EFT may be as effective as the insertion of acupuncture needles. For this reason, the mechanisms of action of acupressure and EFT can be considered the same (Vural and Aslan 2018). Low oestrogen levels seen in the luteal phase cause a decrease in serotonin levels, increasing low mood, stress, anxiety, and fatigue (Jourabchi et al. 2024). Practices such as acupressure and EFT may increase the release of endorphins, enkephalins, and dynorphins, which help reduce pain, and serotonin, which regulates mood. They may also help regulate cortisol levels to more than half their normal levels through the fight-or-flight mechanism, thereby activating the negative feedback mechanism. This regulation of cortisol levels may help relax the central nervous system by reducing pain and slowing the heart rate. In addition, it has been demonstrated that EFT regulates cortisol levels and autonomic nervous system activity (Church and House 2018).

In addition to pharmacological methods that are easy to use and more accessible for reducing symptoms, non-pharmacological methods such as acupressure, yoga, EFT, progressive muscle relaxation exercises, reflexology, massage, and acupuncture are also being utilised. As a result of our research, we found only one study examining the effect of EFT on menstrual symptoms (Sarı Çetin and Erbil 2020; Şimsek Küçükkelepçe et al. 2021; Şimşek Küçükkelepçe and Timur Tashan 2021; Bakır et al. 2022; Coşkun 2024). For this reason, we anticipate that our study will make a significant contribution to the literature.

In our study, the MSS scores and subscale mean scores (Negative/somatic complaints, Menstrual pain, Coping methods) obtained from female students in the experimental and control groups before EFT application were similar, and no statistically significant difference was found (Table 2; *p* > 0.05). In this case, both groups are similar and homogeneous.

In the measurements taken after the EFT application, a significant difference was found between the experimental and control groups in both the MSS score and the subscale mean scores (Table 2; *p* < 0.05). The final test score of the experimental group was measured lower than the pre-test and lower than the final test of the control group (Table 2; *p* < 0.05).

A review of studies utilising the MSS revealed that the mean total and subscale scores were at a moderate level, indicating that menstrual symptoms were commonly experienced among students (Bilgen and İpekçi 2024). Regular exercise and telerehabilitation programmes incorporating different exercises used to improve menstrual symptoms have been found to have a beneficial effect on menstrual symptoms (Akkuş Uçar 2024; Baltaş et al. 2024).

However, a study also found no significant difference between the intervention and control groups in total MSS scale scores and subscale mean scores following application of dry heat to the soles of the feet (Kaya and Açil 2024). Similar results were found in the literature and in our study. In the regression analysis conducted in our study, the model we established was significant, showing that EFT had a reducing effect on menstrual symptoms with a 46.5% change (Table 3; F = 69.530; *p* = 0.000; R^(2)^ = 0.465).

The reduction in menstrual symptom severity with EFT can be attributed to the relaxation of students through touch applied to acupuncture points, specific suggestions, and reminder phrases; the decrease in menstrual pain due to endorphin release; and the improvement in their well-being due to decreased cortisol levels.

In our study, no statistically significant increase in the physical component of the students’ quality-of-life scale was observed following EFT application, whereas a significant increase was observed in the mental component. The regression analysis revealed that EFT positively affected the mental component sub-dimension score by 10.5%. This finding may indicate that EFT positively affects quality of life. EFT may contribute to mental relaxation through touch and positive thinking suggestions. It is thought that the physical component sub-dimension score did not reach the desired level despite alleviation of students’ menstrual pain, due to factors such as their dormitory conditions, eating habits, lifestyles, and school/exam stress. Furthermore, it is thought that this situation arose due to the research’s conditions and timing, the students’ physical well-being and perceptions, and the fact that the final test coincided with exam week. As there are no studies in the literature determining the effect of EFT application on the quality of life of students experiencing PMS throughout the research, the data could not be compared.

Female university students are among the most affected by PMS (Toptaş Acar 2023). Young adolescents experiencing PMS have their mood, self-perception, academic achievement, school/work attendance, work performance, daily and social activities, peer/family relationships, and quality of life negatively affected (Erbil and Yücesoy 2022). According to one study, female university students experiencing PMS concluded that the emotional changes, mental problems, and decreased energy levels they experienced negatively affected their quality of life. Furthermore, they concluded that the methods they chose to alleviate PMS symptoms were insufficient (Topatan and Kahraman 2020). However, it may be suggested that EFT has the potential to positively influence quality of life. (Malik 2021; Lisarni et al. 2022).

No statistical difference was found in SUD scores between the experimental and control groups before the study (Table 2; *p* < 0.05). At the end of the EFT application, a statistical difference was found between the groups, and the students in the experimental group showed a decrease in scores (Table 2; *p* < 0.05). Furthermore, in the regression analysis performed, the model established was significant, and a change of 37.2% was detected (Table 3; F = 47.5966; *p* = 0.000; R^(2)^ = 0.372). Similar to our study, another study found that the SUD scores of the experimental group students decreased significantly compared to the control group students (Bakır et al. 2022). Other studies in the literature using the EFT and SUD scales also show that EFT application reduces SUD scores (Salas et al. 2011; Dincer and Inangil 2021; Bakır et al. 2022; Cici and Özkan 2022). This is thought to be related to the person performing EFT feeling relaxed, increased awareness, and achieving breath control.

The effective use of complementary interventions, such as EFT, by nurses in both clinical practice and educational settings will be an important step toward supporting individuals’ holistic health.

### Limitations

Some limitations of this study should be considered. The data collection process was partially affected by the transition to a hybrid education system following the 6 February 2023 earthquake in the country, which prolonged participant recruitment and follow-up. In addition, the study data were obtained through self-reported scales. Although measures were taken to minimize interaction between groups, it is possible that some participants in the experimental group shared information about the EFT application with participants in the control group during the study process.

## Conclusion

The study findings suggest that the EFT application, administered over two cycles and eight weeks, may reduce premenstrual syndrome symptoms, menstrual symptom severity, and subjective units of distress scale scores. The study findings also suggest that EFT did not produce a significant change in the physical component subscale of quality of life, although it may have contributed to improvements in the mental component subscale. Furthermore, no side effects were encountered during or after the application throughout the study.

In this regard, it is recommended that EFT be taught to nursing students and that their knowledge and awareness of its applications be enhanced. Nurses should be encouraged to participate in EFT certification programmes, where possible, and the integration of this application into clinical practice should be supported. Furthermore, conducting long-term studies involving different age groups and samples, incorporating different EFT rounds, and comparing them with other complementary methods would contribute to the literature.

## Data Availability

No datasets were generated or analysed during the current study.

## Declarations

### Clinical trial number

This study was registered as a clinical trial with the registration number [NCT06557070].
